# Spatial Connectivity and Temporal Dynamic Functional Network Connectivity of Musical Emotions Evoked by Dynamically Changing Tempo

**DOI:** 10.3389/fnins.2021.700154

**Published:** 2021-08-05

**Authors:** Ying Liu, Weili Lian, Xingcong Zhao, Qingting Tang, Guangyuan Liu

**Affiliations:** ^1^School of Mathematics and Statistics, Southwest University, Chongqing, China; ^2^School of Music, Southwest University, Chongqing, China; ^3^College of Preschool Education, Chongqing Youth Vocational and Technical College, Chongqing, China; ^4^School of Electronic and Information Engineering, Southwest University, Chongqing, China; ^5^Faculty of Psychology, Southwest University, Chongqing, China

**Keywords:** dFNC, spatial connectivity, decreasing tempo, increasing tempo, music-evoked emotion

## Abstract

Music tempo is closely connected to listeners’ musical emotion and multifunctional neural activities. Music with increasing tempo evokes higher emotional responses and music with decreasing tempo enhances relaxation. However, the neural substrate of emotion evoked by dynamically changing tempo is still unclear. To investigate the spatial connectivity and temporal dynamic functional network connectivity (dFNC) of musical emotion evoked by dynamically changing tempo, we collected dynamic emotional ratings and conducted group independent component analysis (ICA), sliding time window correlations, and k-means clustering to assess the FNC of emotion evoked by music with decreasing tempo (180–65 bpm) and increasing tempo (60–180 bpm). Music with decreasing tempo (with more stable dynamic valences) evoked higher valence than increasing tempo both with stronger independent components (ICs) in the default mode network (DMN) and sensorimotor network (SMN). The dFNC analysis showed that with time-decreasing FNC across the whole brain, emotion evoked by decreasing music was associated with strong spatial connectivity within the DMN and SMN. Meanwhile, it was associated with strong FNC between the DMN–frontoparietal network (FPN) and DMN–cingulate-opercular network (CON). The paired *t*-test showed that music with a decreasing tempo evokes stronger activation of ICs within DMN and SMN than that with an increasing tempo, which indicated that faster music is more likely to enhance listeners’ emotions with multifunctional brain activities even when the tempo is slowing down. With increasing FNC across the whole brain, music with an increasing tempo was associated with strong connectivity within FPN; time-decreasing connectivity was found within CON, SMN, VIS, and between CON and SMN, which explained its unstable valence during the dynamic valence rating. Overall, the FNC can help uncover the spatial and temporal neural substrates of musical emotions evoked by dynamically changing tempi.

## Introduction

Time seems to exist independently of one’s subjective experience as a physical feature of an objective world. However, our temporal processing is determined by emotions that reflect the upheavals produced in our bodies and minds (Droit-Volet, 2019). Music tempo—as a form of temporal information that arises in response to a musical stimulus—is not purely defined as a stimulus property but also as a psychological experience with multiple evoked emotions (Schirmer et al., 2016). Music tempo is closely connected to listeners’ perception of music emotion. Associated with listeners’ emotional arousal, the change of music tempo was found to be connected with listeners’ sensorimotor synchronization (Hammerschmidt and Wöllner, 2020). Music and movement share a dynamic structure that supports the universal expression of emotions (Sievers et al., 2013), suggesting that music tempo can be a helpful attribute in explaining the neural activities of music-evoked emotion and regulating listeners’ emotion (Ding et al., 2017; Zuk et al., 2018).

Fast music, containing a higher number of events in a given timespan, can engage stronger emotional arousal (excitement or tension) with neural changes in the superior temporal gyrus (STG) and sensorimotor cortex (Nicolaou et al., 2017; Liu et al., 2018). When Jäncke and Sandmann (2010) investigated the influence of background music on verbal learning, they found that even without an enhancing or a detrimental effect on verbal learning performance, compared with slow music, in-tune fast music evoked stronger event-related desynchronization (ERD) around 800–1200 ms after word presentation. Conversely, out-of-tune fast music evoked stronger event-related synchronization for the group exposed to approximately 1600–2000 ms after word presentation. The high-density and high-energy acoustic properties of fast music can arouse more pleasant affective responses with enhanced dissociative attention than slow music (Feiss et al., 2020). Music with a slow tempo evokes sadness or relaxation with a reduction in heart rate (Hilz et al., 2014). Moreover, recent research has found that associated with weak emotional arousal, slow music can enhance the depth of thinking and complex cognitive processing ability of listeners (De Witte et al., 2020). It was also found to enhance the mind-wandering activities associated with DMN (Taruffi et al., 2017). During long-range real-road driving tests, slow-tempo music can temporarily boost a driver’s quality of attention; after an extended period of driving, it significantly deteriorates the driver’s levels of energy and attention (Li et al., 2019).

In daily music-listening activities, music tempi are time-varying, lengthening or shortening a given form of social information by slowing and increasing tempo; this change in tempo, also leads to corresponding changes in emotional meaning (Schirmer et al., 2016). Music-evoked emotion is a typical psychological attribute that can dynamically change alongside music tempo. When a change in music tempo influences a listener’s emotional arousal, researchers found that their music-dependent memory was also affected (Halpern and Müllensiefen, 2008; Kamal and Berry, 2015). Even when rating the emotional content of color stimuli, the faster the presented pieces of music, the happier the color was rated (Tsang and Schloss, 2010). What’s more, these emotional effects of tempo-changing music can also evoke dynamically changing neural activities. A previous study has shown that when the tempo changed on a large scale, it gave rise to a greater change in the α-band spectral power than a smaller tempo change (Ma et al., 2012). ERD in the left motor cortex was most significantly correlated with music tempo changes. Daly et al. (2014) found that ERD strength can be modulated by variations (dynamics) of the music tempo but a piece of music with a steady tempo (either fast or slow) over the duration of the music presentation time does not significantly modulate ERD strength. Functional neuroimaging studies of temporal and emotional processing, as well as studies of brain−damaged patients, have linked components of several cortical and subcortical regions, including the cerebellum, basal ganglia, parietal cortex, prefrontal cortex, premotor cortex, and the supplementary motor area (Rao et al., 2001; Ferrandez et al., 2003; Singer et al., 2016; Koelsch, 2020), which present multiple functional networks in the musical emotion and temporal processing. To clarify the neural substance of emotion evoked in tempo-changing music, it will be of significant value in enhancing the understanding of dynamical emotion and advancing the application of music emotion in clinical therapy. However, the neural mechanism of emotion evoked by music with changing tempi is still unclear.

In order to clarify the spatial connectivity and temporal dynamic activity of musical emotion evoked by dynamically changing tempi, we collected participants’ dynamic emotional ratings and fMRI signals when they were listening to music with decreasing tempo and music with increasing tempo. Dynamic functional neural connectivity was analyzed in a data-driven approach using group independent component analysis (ICA), sliding time window correlations, and k-means clustering. In addition, a paired *t*-test was used to compare the neural differences in the emotions evoked by music presented at each tempo. The current study has an exploratory approach in investigating any significant dynamic functional connectivity between changing tempo and music-evoked emotion. Based on the above neural evidence of temporal processing and musical emotion, we hypothesized that different tempi would elicit different neural activation in the sensorimotor cortex, auditory cortex, and cerebellum (Lin et al., 2014; Harvey, 2020; Sachs et al., 2020). Meanwhile, based on the whole-brain connectivity dynamics in emotions (Ghahari et al., 2020) and the dynamically increasing electroencephalography evidence in music-evoked emotions (Balasubramanian et al., 2018), we hypothesized that functional connections may show significant time-varying changes in SMN, DMN, and FPN.

## Materials and Methods

Forty student volunteers from Southwest University (China) participated in this experiment. Before the experiment, each participant completed the Hamilton Depression Scale. Only those who scored less than eight were invited to participate in the fMRI experiment. Their average age was 20.21 ± 1.33. Twenty of the participants were women. All participants were right-handed native Chinese speakers with normal or corrected-to-normal vision, and none reported a history of neurological disorders, cognitive disability, or use of medications that affect the central nervous system. None of the participants had received professional music training. This study was conducted in accordance with the recommendations of the Southwest University Research Ethics Committee guidelines. All participants provided written informed consent in accordance with the Declaration of Helsinki and were compensated 100 Yuan for their time.

Eight pieces of piano music without lyrics were selected as the experimental materials, while another three pieces of music were selected as training materials. The tempi of the four decreasing musical pieces ranged from approximately 180 to 65 bpm. The tempi of the four increasing musical pieces ranged from approximately 60 to 180 bpm. The details of the eight experiments are presented in Supplementary Table 1. Each musical piece was truncated to a length of 60 s, which was long enough to evoke the desired emotion. The first 2 s of each musical piece faded in, while the last 2 s were faded out to ensure a gentle transition during each session.

### Procedure

**FIGURE 1:**
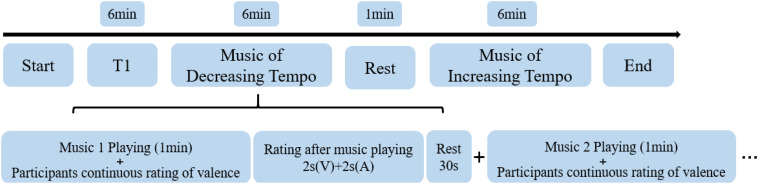
The procedure of fMRI scanning. V, valence; A, arousal.

First, they participated in a training experiment of dynamic emotional rating to familiarize themselves with the emotion ratings. While a piece of music was playing, the participants were asked to rate the valence of each piece as best they could during the 1-min duration from one (negative emotion) to four (positive emotion). When the piece of music ended, they were asked to rate the valence and arousal of music-evoked emotions from one (negative emotion/weak arousal) to four (positive emotion/strong arousal) every 2 s. They were allowed to enter the fMRI scanner only once they had mastered the dynamic scoring requirements. In the fMRI experiment, the participants were asked to rate the valence of four decreasing-tempo pieces and four increasing-tempo pieces and rate their valence/arousal at the end of each piece. The piece in the training experiment did not appear in the formal experiment. A rest period of 30 s was included in-between songs while a rest period of 1 min was included in-between runs. The entire fMRI scanning procedure took approximately 19 min (Figure 1).

After leaving the scanner, the participants completed a second round of rating the emotional valence and arousal of the same eight musical pieces at the end of each song. This score was used as a reference to determine whether the subjects’ emotions differed during the MRI. The post-scanning assessments were completed in a quiet and separate room. The listening procedure was similar to that used for the scanner.

### Data Analysis

Behavioral results were analyzed by multivariate analysis of variance to compare emotional ratings collected both during and after fMRI scanning. Then, paired *t*-tests were used to compare the valence/arousal differences between the scanning and post-scanning assessments by SPSS 20.0. For the dynamic evaluation of valence during music listening, the number of evaluation and standard deviations (SD) were computed to describe the valence characteristics of decreasing-tempo and increasing-tempo.

Images were acquired using a Siemens 3T scanner (Siemens Medical Systems, Erlangen, Germany). An echo-planar imaging sequence was used for image collection, and T2-weighted images were recorded in each run. T1-weighted images were collected to yield a total of 176 slices at a thickness of 1 mm and an in-plane resolution of 0.98 mm × 0.98 mm.

Functional images were preprocessed using SPM8 Preprocessing, which included the removal of the first three image volumes to avoid T1 equilibration effects, realignment using INRI align, slice-timing correction using the middle slice as the reference frame, spatial normalization into Montreal Neurological Institute (MNI) space, re-slicing to 3 mm^3^ voxels, and smoothing with a Gaussian kernel. The full width at half maximum was specified as 4 mm^3^. Then, we obtained six direction parameters for head movement. We deleted the data of participants with head movement of more than 2.5 mm in any direction. After preprocessing, data of thirty-eight participants were kept for further analysis.

Group ICA, sliding time window correlations, and k-means clustering were all performed using fMRI toolbox (GIFT) software. Independent component analysis, was used to extract spatially independent but temporally coherent components. In the session of each music group, data sets were temporally concatenated through participants. The data dimensions were reduced to the number of ICs using principal component analysis (PCA). The optimal number of ICs in the dataset was 23 for decreasing-tempo music and 25 for increasing-tempo music. Accordingly, the Infomax algorithm was used to decompose the data from all participants. To determine the repeatability of the ICs, 150 IC iterations were performed using ICASSO (a tool for reliability investigation of independent component analysis estimates). The individual IC maps and time courses were computed via backward reconstruction using both aggregate components and the results from the data reduction step. Functional network connectivity (FNC) correlations averaged over participants at each music tempo were analyzed using one sample *t*-tests (*t* = 1). All components were marked based on the Dosenbach-160 regional atlas.

Subsequently, dynamic FC was estimated using a sliding window approach. We used a tapered window created by convolving a rectangle (width = 30 TRs = 60 s) with a Gaussian (σ = 3 TRs) and sliding in steps of 1 TR, resulting in 148 windows. Because relatively short time segments may have insufficient information to characterize the full covariance matrix, we estimated covariance from the regularized precision matrix. In accordance with the graphical least absolute shrinkage and selection operator method of Friedman et al. (2008), we placed a penalty on the L1 norm of the precision matrix to promote sparsity. The regularization parameter lambda (λ) was optimized separately for each participant by evaluating the log-likelihood of unseen data (windowed covariance matrices from the same participant) in a cross-validation framework. Final dynamic FC estimates for each window were concatenated in covariance (correlation) between components as a function of time. The dynamic FC estimates were Fisher transformed to stabilize the variance before further analysis.

The k-means clustering algorithm was then applied to assess the frequency and structure of reoccurring FC patterns to windowed covariance matrices by the square Euclidean distance. The clustering algorithm was applied to the set of all participant exemplars and was repeated 500 times to increase the chances of escaping local minima. The maximum number of iterations was 1,000. The resulting centroids (cluster medians) were then used to initialize the clustering of all data. Both methods produced clusters that were almost identical to those observed using windows at local maxima. Likewise, we repeated clustering using different distance functions (correlation, city-block, and cosine) and found extremely similar results. For group clustering, the number of clusters (k) was determined using the elbow criterion of the cluster validity index, computed as the ratio of within-cluster distance to between-cluster distance, although additional exploratory analyses using hierarchical clustering or explicit variations in k demonstrated consistent results over a large range of k.

After the dynamic FNC analysis, paired *t*-tests were used to analyze the neural differences associated with the emotions evoked by the two types of music. Thirty-eight pairs were compared in decreasing-increasing condition. The threshold was false discovery rate (FDR)-corrected, and p-values < 0.05 were considered significant in univariate analyses.

## Results

### Behavioral Results

**FIGURE 2:**
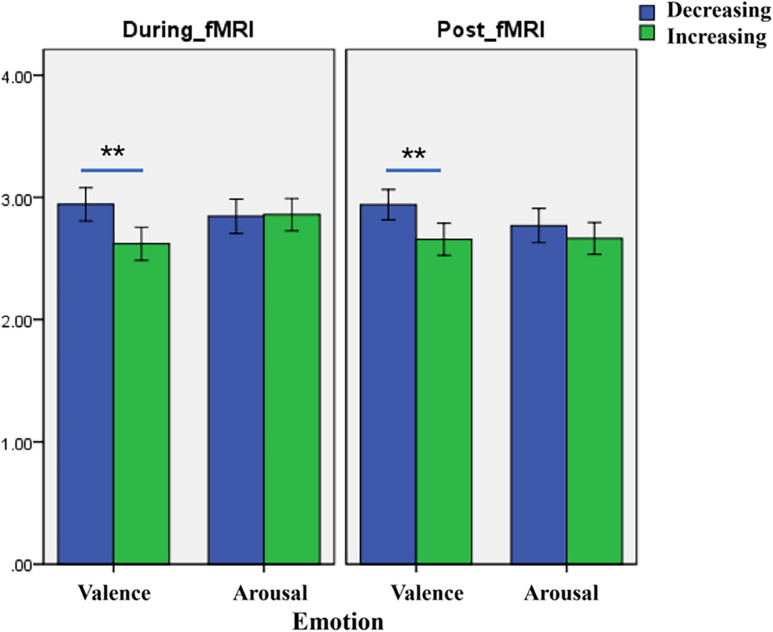
The difference of valence between decreasing music and increasing music (** means *p* < 0:01), which presents a significant difference between the two groups of behavioral data and can occur 99%.

**FIGURE 3:**
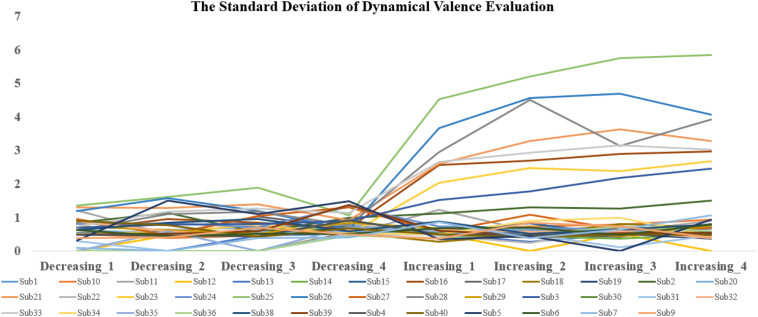
The distribution of all participants’ rating SD for all songs.

The analysis revealed significant main effects of valence both during and after the scans. The difference in fMRI valence was significant both during fMRI scanning [*p* < 0.01, *F* (1, 302) = 11.02, Eta^2^ = 0.04] and post-fMRI scanning [*p* < 0.01, *F* (1, 303) = 9.63, Eta^2^ = 0.03] (Table 1 and Figure 2). The difference in arousal was not statistically significant. No difference was found in valence between the post-rating and the fMRI-rating. The dynamic evaluation of valence during the music listening process is presented in Table 2. A significant difference was found in the rating scores and SD between the decreasing-tempo and increasing-tempo pieces of music. The pieces of music with increasing tempi yielded a higher rating (*t* = 3.11, *p* < 0.01) and bigger SD (*t* = 4.98, *p* < 0.01) than the pieces of music with decreasing tempi. Table 3 presents the details of the rating scores and SD of the eight songs. Figure 3 presents the distribution of all participants’ rating SD for all the songs. We compared the mean valence of the front segment and back segment by dividing the dynamic ratings into two sections. In pieces with decreasing tempi, the valence changed from 2.79 ± 0.23 to 2.81 ± 0.61 (*p* > 0.05). In music with increasing tempi, the valence changed from 2.53 ± 0.71 to 2.72 ± 1.33 (*p* < 0.05).

**TABLE 1:** The ratings of valence and arousal evoked by music.

		Decreasing tempo	Increasing tempo	Significance(p)	Eta^2^
During-fMRI	Valence	2.94 ± 0.81	2.62 ± 0.81	0.001	0.038
	Arousal	2.84 ± 0.86	2.86 ± 0.81	0.890	0.00
Post-fMRI	Valence	2.94 ± 0.77	2.66 ± 0.81	0.002	0.031
	Arousal	2.77 ± 0.87	2.66 ± 0.81	0.277	0.004

**TABLE 2:** The dynamic evaluation of valence during music listening.

	N	Average	SD	T	Correlations
Decreasing_Num	152	59.89	45.43	−3.13**	0.77**
Increasing_Num	152	68.04	44.33		
Decreasing_SD	152	0.66	0.37	−4.98**	0.05
Increasing_SD	152	1.19	1.25		

** Means *p* < 0.01.

**TABLE 3:** The rating number and SD of each song.

Music	N	Number of evaluation				SD of evaluation			
		Minimum	Maximum	Average	SD	Minimum	Maximum	Average	SD
Decreasing_1	38.00	3.00	159.00	63.00	47.43	0.00	1.36	0.58	0.35
Decreasing_2	38.00	2.00	136.00	56.16	42.75	0.00	1.62	0.64	0.42
Decreasing_3	38.00	2.00	154.00	61.39	47.30	0.00	1.89	0.66	0.40
Decreasing_4	38.00	2.00	157.00	59.00	45.64	0.40	1.48	0.76	0.28
Increasing_1	38.00	4.00	185.00	72.32	45.76	0.27	4.53	1.06	1.02
Increasing_2	38.00	1.00	179.00	67.76	47.89	0.00	5.20	1.21	1.31
Increasing_3	38.00	4.00	177.00	65.95	43.47	0.00	5.76	1.18	1.33
Increasing_4	38.00	4.00	172.00	66.13	41.42	0.00	5.85	1.25	1.31

### Dynamic FNC Results

#### ICA of Emotion Evoked by Music With Decreasing and Increasing Tempo

**FIGURE 4:**
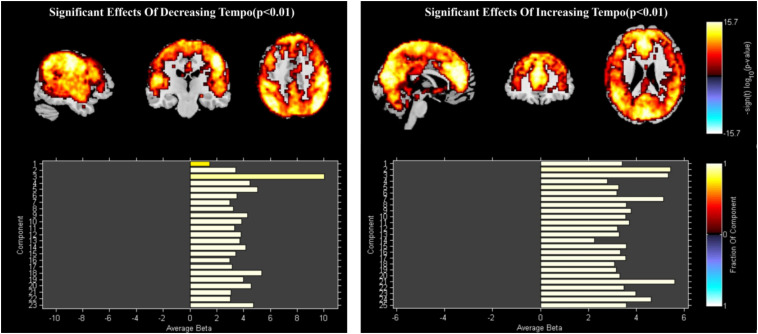
The group ICs of decreasing tempo and increasing tempo.

Data from 38 participants were included in the dFNC analysis. PCA identified 23 ICs. IC1 and IC3 were deleted because of their scattered and weak activation, resulting in a total of 21 ICs for further analysis. For the increasing-tempo condition, PCA identified 25 ICs (Figure 4). Based on their anatomical and presumed functional properties, the ICs were arranged into the following groups: DMN, FPN, CON, SMN, VIS, CB, and auditory (AUD). The global distribution of the whole brain is presented in Figure 4 and the coordinate information of ICs is presented in Supplementary Table 2.

#### Paired *t*-Test of the Group ICs

**FIGURE 5:**
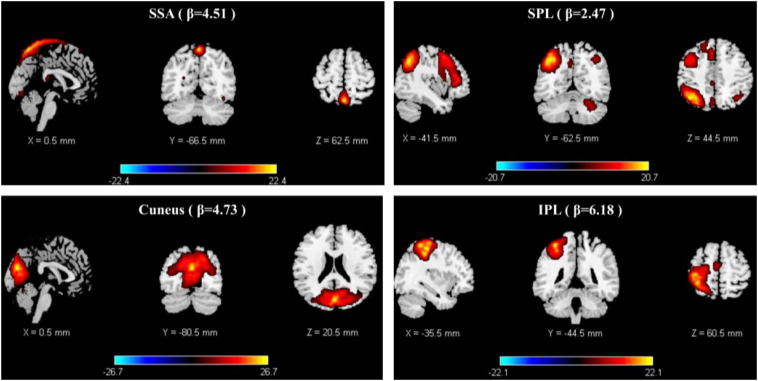
The stronger activation of ICA in decreasing music than increasing music.

Paired *t*-tests were performed using FDR correction (*p* < 0.05). When examining the difference between decreasing and increasing-tempo music, we identified 23 ICs and observed significantly stronger activations in the DMN (Cuneus) and SMN (SSA, IPL, SPL) (Figure 5).

### FNC of Emotion Evoked by Music With Decreasing and Increasing Tempo

**FIGURE 6:**
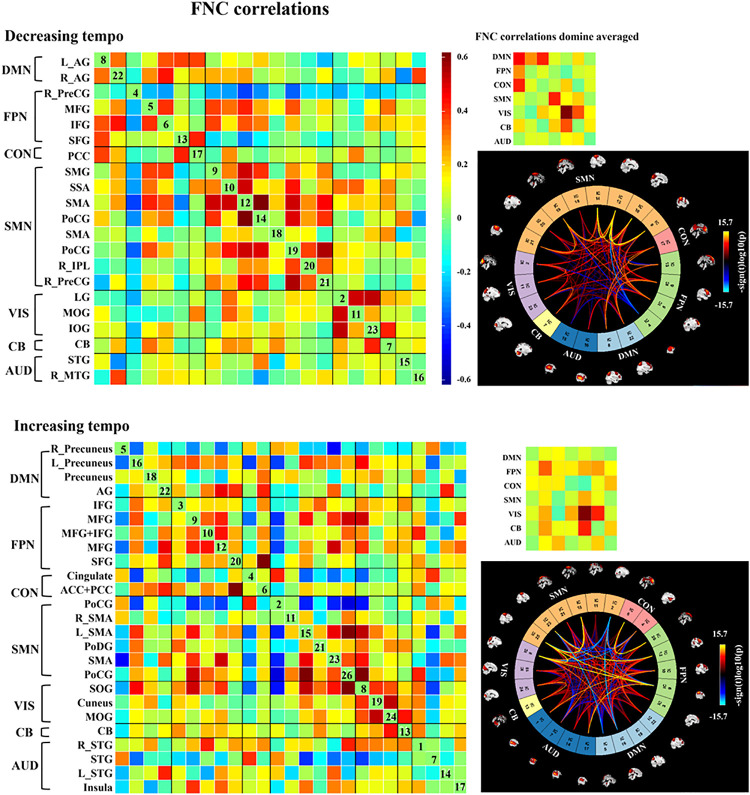
Group ICA in the two kinds of music. In each level, the left matrix displays the FNC correlations averaged over subjects. In the upper right corner, the small matrix presents the correlations averaged in each network. In the bottom right, the connectogram displays the significant effects when *p* < 0.001, and the distribution of the color bar means the T score. AG, angular gyrus; IFG, inferior frontal gyrus; PFC, prefrontal cortex; dlPFC, dorsal lateral prefrontal cortex; MFG, middle frontal gyrus; IPL, inferior parietal lobe; SPL, superior parietal lobe; PoCG, postcentral gyrus; PreCG, precentral gyrus; SMA, sensorimotor area; IOG, inferior occipital gyrus; MOG, middle occipital gyrus; LG, lingual gyrus; PCC, posterior cingulate cortex; CB, cerebellum; SOG, superior occipital gyrus; STG, superior temporal gyrus; MTG, middle temporal gyrus; SAA, somatosensory association area; SAA, sensory association area; SFG, superior frontal gyrus; TTG, transverse temporal gyrus; TL, temporal lobe.

We observed three notable FNC features that differed from the emotions evoked by the music presented at two tempi. First, decreasing-tempo music evoked stronger FNC than increasing-tempo music, especially when referring to the averaged connections of DMN, CON, and SMN. Second, increasing-tempo music evoked the stronger FNC of FPN. Third, strong activation within VIS and between VIS-CB were found in music with both tempi (Figure 6).

### Dynamic FNC of Emotion Evoked by Music

**FIGURE 7:**
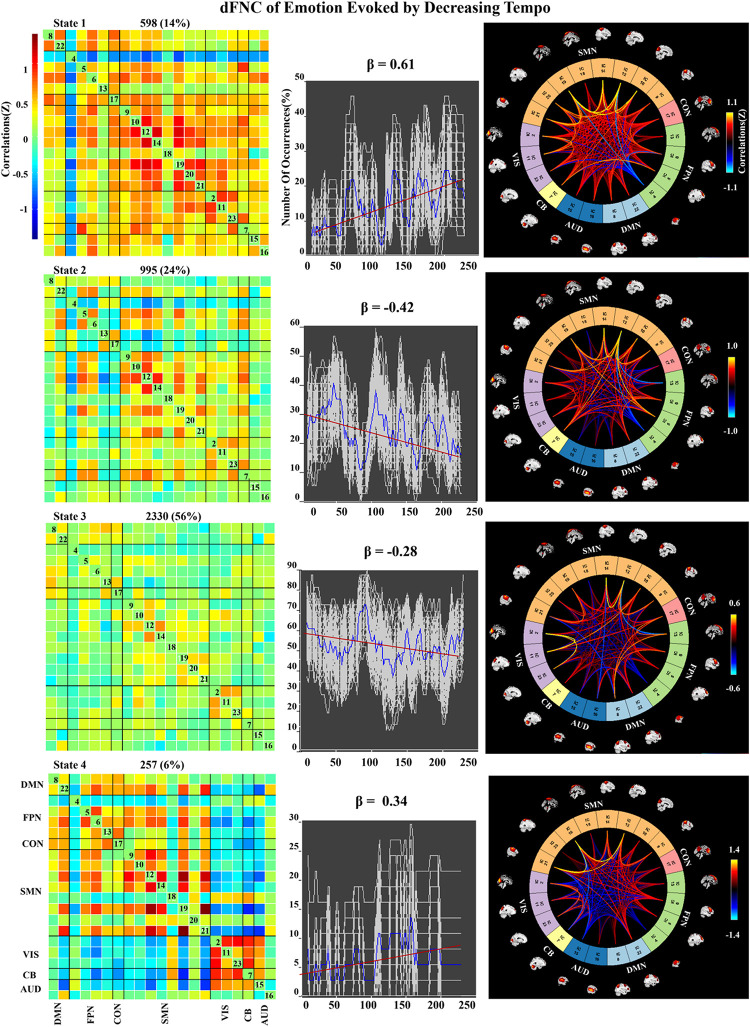
Correlation matrix, number of occurrences, and connectograms of four clusters of evoked emotion by decreasing -tempo music.

**FIGURE 8:**
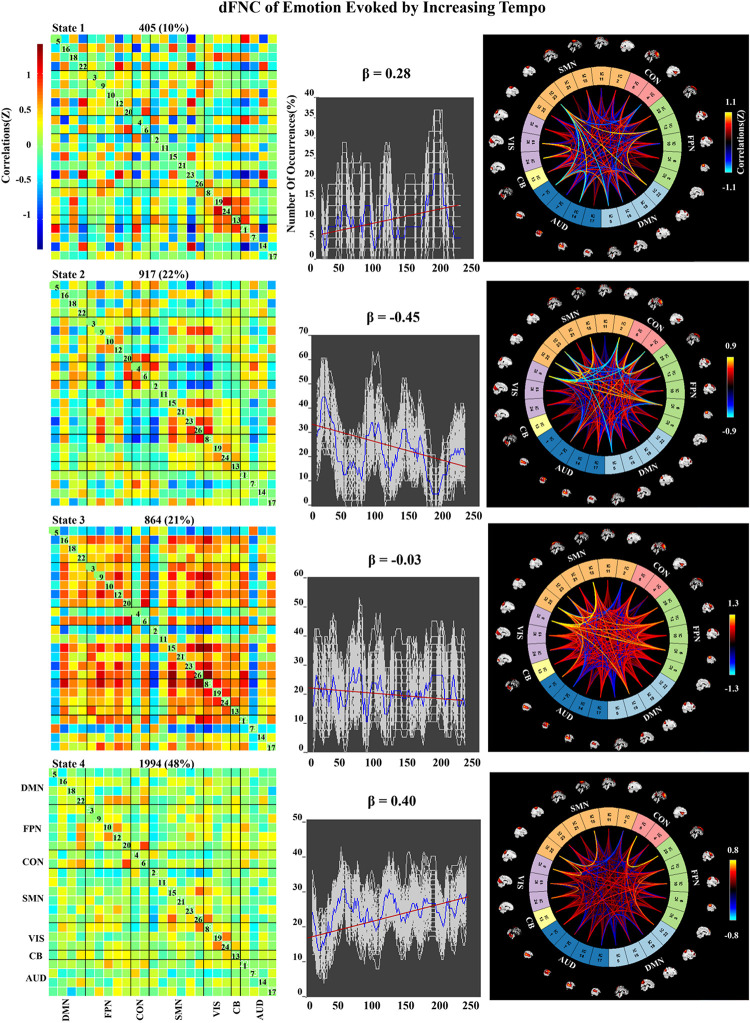
Correlation matrix, number of occurrences, and connectograms of four clusters of evoked emotion by increasing -tempo music.

We applied k-means clustering to the windowed FC matrices to explore the possibility that certain connectivity patterns may be quasi-stable; that is, that they reoccur over time and are present in numerous participants. The clustering resulted in *k* = 4 across all two groups. The cluster centroids and connectograms of the emotions evoked by decreasing and increasing-tempo music are separately displayed in Figures 7, 8. In these two figures, each matrix represents the centroid of a cluster and putatively reflects a stable connectivity state within the data.

In the decreasing-tempo condition (Figure 7), State 3 accounted for 56% of all windows based on our observations from higher-order and hierarchical clustering, signifying that the brain networks in this connected state play a leading role in the entire dynamic process. Meanwhile, State 3 showed a changing trend of decreasing connectivity (β = −0.28), which showed that the network connectivity of evoked emotions in the brain decreased with changes in music tempi. State 2 accounted for 24% of all windows and showed a stronger decreasing trend with significant connectivity within SMN and VIS and between SMN–VIS (β = −0.42), which provided the evidence of attenuated engagement of sensorimotor processing and visual processing when the tempo changed from fast to slow.

In the increasing-tempo condition (Figure 8), State 4 accounted for 48% of all windows and showed an increasing trend of whole brain connectivity (β = 0.40). The increasing tempo can engage increasing neural activities in the whole brain of listeners. However, State 2 accounted for more than 22% with decreasing network connectivity (β = −0.45) with SMN and SMN–CON. As an important neural area of emotional process, the decreasing SMN and SMN-CON can be evidence of bigger emotional fluctuations when the tempo changed from slow to fast.

All subjects’ state transitions for the two kinds of music are presented in Supplementary Figure 1.

## Discussion

In this study, we explored the spatial and temporal FNC of emotions evoked by music presented at dynamically changing tempi using group ICA, sliding windows, clustering, and paired *t*-tests. By comparing the different spatial connectivity and temporal dynamics between decreasing and increasing-tempo music, our analysis provides the first behavioral relationship between changing tempi and their emotional effect with supporting whole-brain characterization of regional differences in FC variability and distinction of discrete FC states. These results and their implications are discussed with regard to spatial connectivity and temporal dynamics.

### The Spatial Connectivity of SMN and DMN in Emotion Evoked by Music With Decreasing- and Increasing-Tempo

Changes in music tempi are closely related to listeners’ emotional processes. In the 1-min music listening task, the mean tempo of decreasing-tempo music (180–65 bpm) and increasing-tempo music (60–180 bpm) was similar and the musical events of the two pieces of music should be close. In the current study, music with decreasing tempo showed significantly higher valence than music with increasing tempo both during and after fMRI scanning. The higher valence of the decreasing-tempo music indicated that the fast tempo at the beginning of the musical pieces delivered more pleasure than the fast tempo at the end, which may have engaged more attention or sensorimotor neural activation (Shih et al., 2009; Wallengren and Strukelj, 2015). Indeed, the paired *t*-test of group ICA showed that decreasing-tempo music activated stronger functional neural connectivity than increasing-tempo music in the bilateral SSA, left SPL, left IPL, and bilateral cuneus. The SSA, SPL, and IPL are important neural areas of the SMN. The cuneus belongs to the DMN. When analyzing the functional network connectivity of music-evoked emotions, decreasing-tempo music evoked stronger activation within the SMN and DMN and between DMN–FPN and DMN–CON than with increasing-tempo music. SMA has been found to participate in the sensorimotor synchronization and emotional processing of fast music (Krause et al., 2010; Ren et al., 2019; Clayton et al., 2019; Proverbio et al., 2020). Even in 8-year-old children, their better performance in emotion recognition can be predicted by stronger connectivity between the inferior frontal gyrus and motor regions, including the primary motor, lateral premotor, and supplementary motor sites (Correia et al., 2019). The enhanced activation of the ICs in the SMN and its stronger functional connectivity provided neural evidence of the higher valence evoked by decreasing-tempo music.

The DMN contains key regions that are recruited in social cognitive emotion regulation (Xie et al., 2016; Satpute and Lindquist, 2019). As the main IC of DMN in decreasing-tempo music, the bilateral AG has been proven to be associated with emotional regulation (Kohn et al., 2014), which even showed enhanced functional connectivity in imagined music performance (Tanaka and Kirino, 2019). In the current study, a stronger FC was found not only within DMN but also between DMN–FPN and DMN–CON. Frontoparietal network is an important neural area for working memory and attention to external stimuli (Fernandez et al., 2019; Wang et al., 2020). The enhanced functional connectivity within the DMN and between DMN–FPN and DMN–CON can be evidence of the emotional regulation evoked by decreasing-tempo music. Combined with the stable valence evaluations made during the music listening process, the interactive relationship between DMN and FPN indicated that the brain may invoke neural functional networks of deep thinking and cognitive controlling (Allen et al., 2014) to coordinate listeners’ emotional experiences as the tempi transition from fast to slow. Moreover, decreasing-tempo music evoked stronger FNC in CON than increasing-tempo music. The cingulate cortex is an indispensable neural structure for emotional processing in music (Koelsch, 2018, 2020). Tanaka and Kirino (2019) found that the PCC had enhanced connections with the AG during imagined music activities. In the current study, bilateral AG was the main ICA of DMN and PCC was the main ICA of CON, which supported the higher valence and more stable emotion processing in decreasing-tempo music.

### The Temporal Dynamics FNC of Music Evoked by Music With Decreasing- and Increasing-Tempo

In the dynamic evaluation of valence, music with increasing tempo evoked a larger SD than decreasing-tempo music. When we divided the dynamic ratings of valence into front segment and back segment, the valence increased from 2.53 ± 0.71 to 2.72 ± 1.33 with a significant difference (*p* < 0.05), which suggested that the emotion was more fluctuant when evoked by a change in tempo from slow to fast. In the current study, the neural activation of SMN and DMN was weaker in increasing-tempo music, as evidenced by the paired *t*-test (Figure 5). The FNC of the whole brain was also lower in the average FNC correlation of increasing music (Figure 6). Less brain activity is not conducive to maintaining the emotional stability of the brain. Although with increasing FNC in the emotion evoked by increasing-tempo music (Figure 8, state 4), state 2 (22%) of increasing-tempo music showed a decreasing change with SMN and SMN–CON. As an important neural area of emotional process, the decreasing SMN and SMN-CON in increasing-tempo music can provide evidence of bigger emotional fluctuation when the tempo changes from slow to fast. Compared with decreasing-tempo music, the slow tempo at the beginning of the increasing-tempo pieces was weak in arousing listening attention and emotional experiences, it can be speculated that the unstable emotional valence of increasing music was associated with fewer engagement of neural processing resources (Vigliocco et al., 2014).

In the dynamic FNC of increasing-tempo music, the principle state also showed a larger variation tendency (Figure 8, state 4, β = 0.4) than that of decreasing-tempo music (Figure 7, state 3, β = −0.28). These dynamic results showed that the change in the listeners’ brain connectivity was consistent with the change in tempo. When the tempo transitioned from fast to slow, the FNC of the principle state (54%) decreased with evenly distributed connectivity across the whole brain. In contrast, when the tempo changed from slow to fast, the FNC of the principle state (48%) increased across the whole brain. As the first dFNC evidence of tempo-changing music, this finding intuitively shows the fundamental activities of music-evoked emotions in dynamic processing. In the second-largest state of decreasing-tempo music, the decreasing FNC of SMN (β = −0.42) indicated that listeners’ sensorimotor processes may lose superiority when music slows down, which was consistent with the change in the principle state. However, the second-largest state of increasing-tempo music was the opposite of the change in the principle state. We speculated that in music listening, early emotional arousal and pleasure are more conducive to keeping listeners’ emotions in a stable state. In cognitive or emotional tasks, the temporal dynamics of neural activity changing with the music tempo is an important aspect of further exerting its psychological regulatory function and helpful in revealing the neural substance of human emotion processing.

### FNC of VIS and CB for Emotions Evoked by the Changing-Tempo Music

In the averaged FNC correlation of these two kinds of music, both activated the strong functional connectivity within VIS and between VIS–CB. Music is an intriguing stimulus to evoke vivid and soothing visual imagery (Aleman et al., 2000). When illuminating the neural mechanism of music-evoked emotion, Juslin and Västfjäll (2008) highlighted that visual imagination is fundamental in inducing musical emotion. During natural music listening activities, a k-means cluster analysis revealed 10 clusters referring to brain areas typically involved in music and emotional processing, namely in the proximity of thalamic-limbic and orbitofrontal regions as well as in the frontal, fronto-parietal, parietal, parieto-occipital, temporo-occipital, and occipital areas (Rogenmoser et al., 2016). In the current study, the enhanced FNC of VIS (occipital area) was consistent with the neural activities of music-evoked emotion. The cerebellum has been found to be involved in emotion processing when music is used in movies to increase audiences’ visual–emotional experiences (Baumgartner et al., 2006) and regulate listeners’ sensorimotor synchronization (Van Der Steen and Keller, 2013; Comstock et al., 2018). In the current study, the enhanced FNC of CB evoked by two kinds of music can be helpful to listeners’ emotional processing and sensorimotor synchronization during performances of dynamically changing music.

## Limitations

This study addressed the spatial connectivity and temporal dynamics of functional networks associated with emotions evoked by the music presented at a dynamically changing tempo. However, the current study needs to be improved due to the absence of signal synchronization of music tempo, emotional ratings, and neural responses. In future studies, a new experimental paradigm may be used to provide more direct evidence about dynamic neural activities. Besides, further research will balance the order of decreasing-tempo and increasing-tempo randomly and collect data of musicians to improve the ecological validity of the research.

## Conclusion

By using the data-driven approach of group ICA, sliding time window correlations, and k-means clustering, we explored the spatial connectivity and temporal dynamic FNC of emotion evoked by music with dynamically changing tempi, which shows a close correlation to multifunctional neural networks. Music with decreasing tempo can evoke enhanced neural networks in emotional processes to keep listeners in a stable state of pleasure with enhanced FNC in DMN, SMN, and FPN. Music with increasing tempo was weak in arousing multiple neural networks, which resulted in listeners’ unstable emotion processing. VIS and CB are thus both important in the emotional processing of decreasing and increasing-tempo music. In future, more work in FNC is needed to explain the dynamic neural mechanism of music-evoked emotion.

## Data Availability Statement

The original data for this study can be found online at: https://pan.baidu.com/s/1vsh_Ic-Ln2s11iVOsmLPRw (Code: LYTM).

## Ethics Statement

The studies involving human participants were reviewed and approved by the Ethics Committee, Faculty of Psychology, Southwest University. The patients/participants provided their written informed consent to participate in this study.

## Author Contributions

YL and GL were responsible for the experimental design and manuscript writing. WL was responsible for the language polishing. XZ and QT were responsible for data collection and analyzing the data. All of the authors have read and approved the manuscript.

## Conflict of Interest

The authors declare that the research was conducted in the absence of any commercial or financial relationships that could be construed as a potential conflict of interest.

## Publisher’s Note

All claims expressed in this article are solely those of the authors and do not necessarily represent those of their affiliated organizations, or those of the publisher, the editors and the reviewers. Any product that may be evaluated in this article, or claim that may be made by its manufacturer, is not guaranteed or endorsed by the publisher.

## Supplementary Material

Supplementary Figure 1 All subjects’ state transitions for the two kinds of music.

Supplementary Table 1 The details of the experiment music.

Supplementary Table 2 The coordinate information of ICs.
