# Supplementary material for: Spatial Connectivity and Temporal Dynamic Functional Network Connectivity of Musical Emotions Evoked by Dynamically Changing Tempo
Source: Front Neurosci. 2021 Aug 5;15:700154. doi: 10.3389/fnins.2021.700154 (PMC8375772; doi:10.3389/fnins.2021.700154)
Supplement: Supplementary Figure 1 — All subjects’ state transitions for the two kinds of music. [file Data_Sheet_1.docx]

Supplementary Figure 1. All subjects’ state transitions for the two kinds of music.


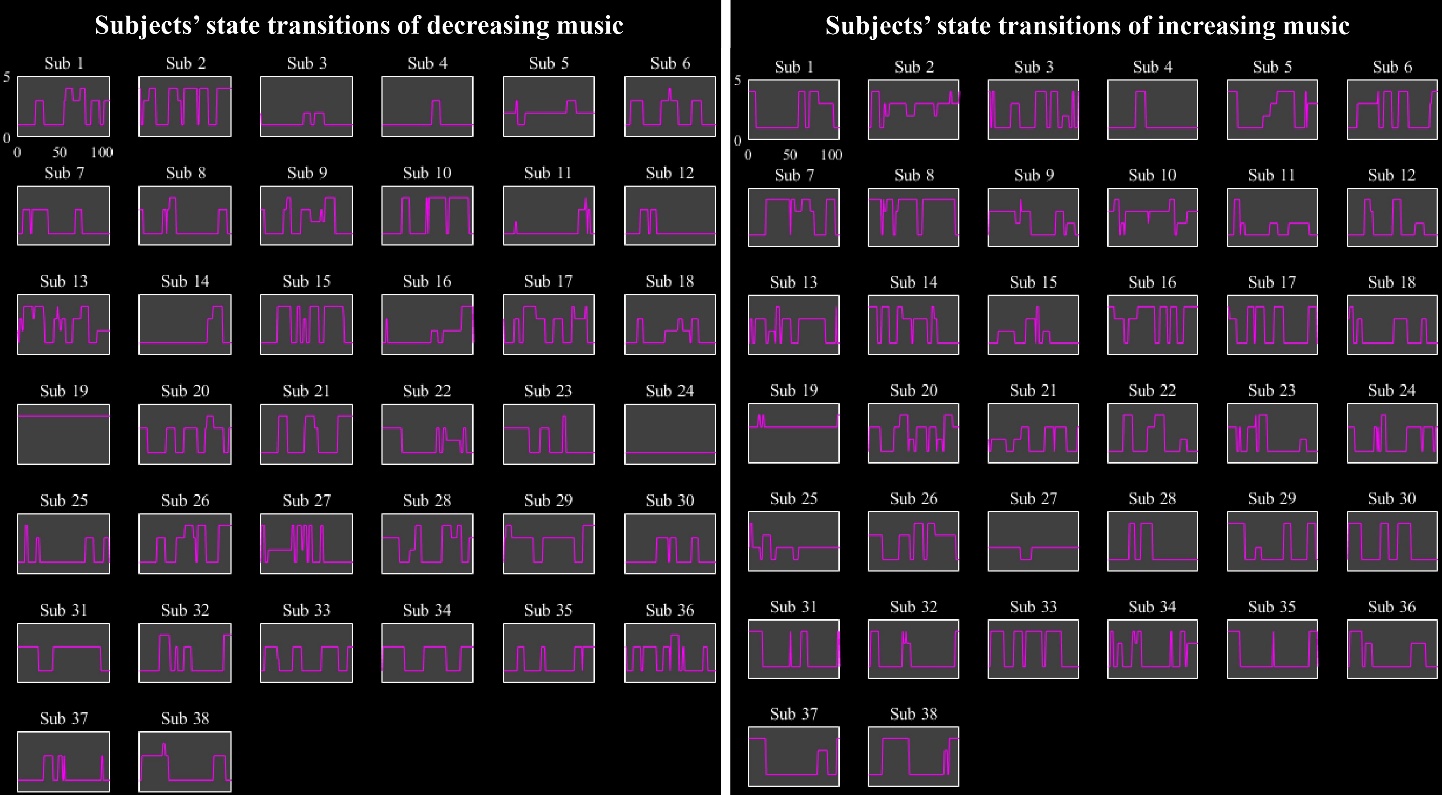


Supplementary Table 1. The details of the experiment music

|  | **Tempo** | **Music** | **Edition** | **bpm** |
| --- | --- | --- | --- | --- |
| 1 | Decreasing | Czerny, 599-63 | Erster Lehrmeister | 140-80 |
| 2 |  | Trudeau's Dream | Lang Lang | 125-65 |
| 3 |  | Carmen overture | Classical Day Bank | 180-70 |
| 4 |  | Sinfonia of Spring Festival Ensemble-1 | Lang Lang | 165-70 |
| 8 | Increasing | Chinese Cowboy With His Flute | Lang Lang | 90-135 |
| 9 |  | Humor with sorrow | [Beethoven](http://www.ktvc8.com/230601.html) | 65-180 |
| 10 |  | North Wind Blow | Chengzong Yin | 60-170 |
| 11 |  | Partita of little stars | Mozart | 70-120 |

| **ICs in Decreasing** | **x** | **y** | **Z** | **ICs in Increasing** | **x** | **y** | **z** |
| --- | --- | --- | --- | --- | --- | --- | --- |
| 2 | 2.5 | -80.5 | 23.5 | 1 | 54.5 | -51.5 | 15.5 |
| 4 | 59.5 | 15.5 | 9.5 | 2 | 38.5 | -30.5 | 71.5 |
| 5 | 30.5 | 53.5 | 23.5 | 3 | -33 | 47.5 | 29.5 |
| 6 | -47.5 | 8.5 | 23.5 | 4 | 33.5 | 15.5 | -8.5 |
| 7 | -17.5 | 75.5 | -21.5 | 5 | 23.5 | -78.5 | 36.5 |
| 8 | -35.5 | -69.5 | 39.5 | 6 | -3.5 | 44.5 | 12.5 |
| 9 | 56.5 | -32.5 | 44.5 | 7 | -57.5 | 11.5 | 26.5 |
| 10 | 3.5 | -71.5 | 39.5 | 8 | 23.5 | -77.5 | 42.5 |
| 11 | 29.5 | -80.5 | 21.5 | 9 | 59.5 | -36.5 | 33.5 |
| 12 | -3.5 | 69.5 | 56.5 | 10 | 47.5 | -44.5 | 47.5 |
| 13 | -15 | 51 | 42 | 11 | 54.5 | -6.5 | 36.5 |
| 14 | -32.5 | -33.5 | 51.5 | 12 | -47.5 | 11.5 | 33.5 |
| 15 | -54.5 | -18.5 | 14.5 | 13 | 23.5 | -71.5 | -27.5 |
| 16 | 56.5 | -5.5 | -12.5 | 14 | -56.5 | -33.5 | 5.5 |
| 17 | 2.5 | -57.5 | 24.5 | 15 | -15.5 | -39.5 | 68.5 |
| 18 | 0.5 | -66.5 | 62.5 | 16 | -2.5 | -71.5 | 33.5 |
| 19 | 23.5 | -33.5 | 68.5 | 17 | 45.5 | -12.5 | 11.5 |
| 20 | 30.5 | -44.5 | 60.5 | 18 | -35.5 | -74.5 | 32.5 |
| 21 | 51.5 | -17.5 | 36.5 | 19 | 2.5 | -80.5 | 18.5 |
| 22 | 47.5 | -62.5 | 41.5 | 20 | -3.5 | 41.5 | 53.5 |
| 23 | 29.5 | -87.5 | 3.5 | 21 | 2.5 | -66.5 | 62.5 |
|  |  |  |  | 22 | -41.5 | -63 | 43 |
|  |  |  |  | 23 | -35.5 | -44.5 | 57.5 |
|  |  |  |  | 24 | 26.5 | -87.5 | -2.5 |
|  |  |  |  | 25 | 38.5 | -35.5 | 54.5 |

Supplementary Table 2. The coordinate information of ICs.
